# Case report of Modified Viabahn Open Revascularization TEChnique (VORTEC) as a rescue strategy for hepatic artery dissection after initial endovascular treatment of postpancreaticoduodenectomy hemorrhage

**DOI:** 10.1097/MD.0000000000029176

**Published:** 2022-05-20

**Authors:** Yi Ping Sng, Zhihao Li, Hsu-Ting Yen, Chee Chien Yong

**Affiliations:** a Department of General Surgery, Kaohsiung Chang Gung Memorial Hospital, Kaohsiung, Taiwan; b Department of Cardiovascular Surgery, Kaohsiung Chang Gung Memorial Hospital, Kaohsiung, Taiwan.

**Keywords:** endovascular intervention, hepatic artery dissection, postpancreaticoduodenectomy hemorrhage, Viabahn, Viabahn Open Revascularization TEChnique, Whipple surgery

## Abstract

**Rationale:**

Currently endovascular treatments are commonly utilized to treat postpancreaticoduodenectomy hemorrhage. However, when endovascular procedure went wrong, open surgery with ligation of the culprit vessels would be the most common salvage method. With Modified Viabahn Open Revascularization TEChnique (VORTEC), we can try to rescue the vessel without sacrificing it by introduction of another endovascular stent under direct method.

**Patient concerns:**

A 76-year-old man with stage IIIA ampulla vater adenocarcinoma underwent pancreaticoduodenectomy and experience pancreatic leak complicated with postpancreaticoduodenectomy hemorrhage.

**Diagnosis:**

Emergent angiography revealed extravasation from proper hepatic artery.

**Interventions:**

A 6 mm Viabahn stent was deployed but no distal runoff. Operation was shifted to emergent laparotomy and revealed intimal dissection of hepatic artery. Modified VORTEC was performed with guidewire redirected to true lumen and another stent was deployed under direct vision.

**Outcomes:**

Patient's hepatic artery was preserved and with no consequent liver failure.

**Lesson:**

Modified VORTEC method could be used as salvage strategy for artery dissection after initial endovascular treatment failed.

## 1 Introduction

Pancreaticoduodenectomy is a complex surgery with 30% to 40% morbidity and 5% mortality, where its early complications include sepsis, anastomosis leak with fistula formation, and hemorrhage. Postpancreaticoduodenectomy hemorrhage (PPDH) is seen in less than 10% of patient but mortality up to 11% to 50%.^[1]^ In recent years, endovascular treatment is widely used for bleeding control with optimal outcome but if it failed, laparotomy is considered last resort. However, introduction of Viabahn Open Revascularization TEChnique (VORTEC) provides another possible solution for bleeding control without compromising blood flow. VORTEC was initially used in complex aortic hybrid surgeries and we modified it to salvage dissected arteries.

## 2 Case presentation

**Figure 1:**
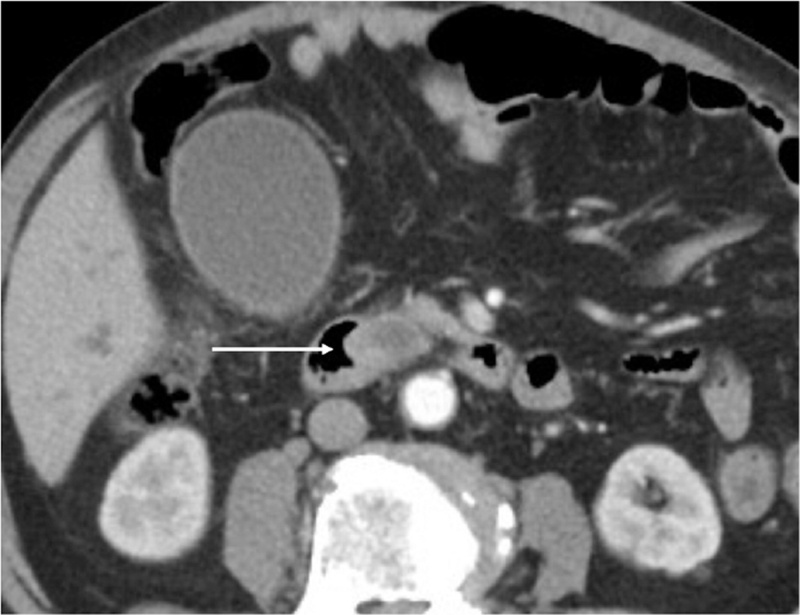
Arrow: CT revealed a 2.16 × 1.92 cm enhancing nodule over ampulla vater region. CT = computed tomography.

**Figure 2:**
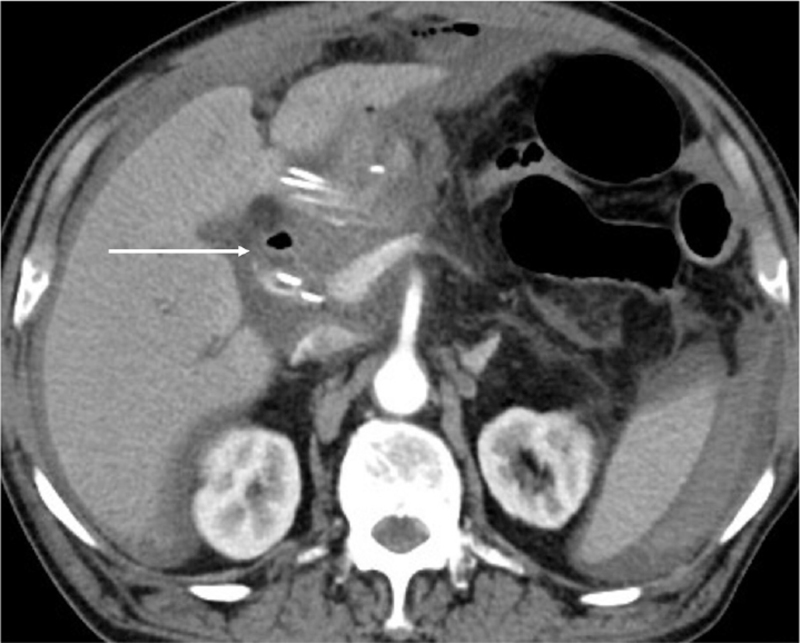
Arrow: CT angiography showed hemoperitoneum with no active extravasation of contrast. CT = computed tomography.

**Figure 3:**
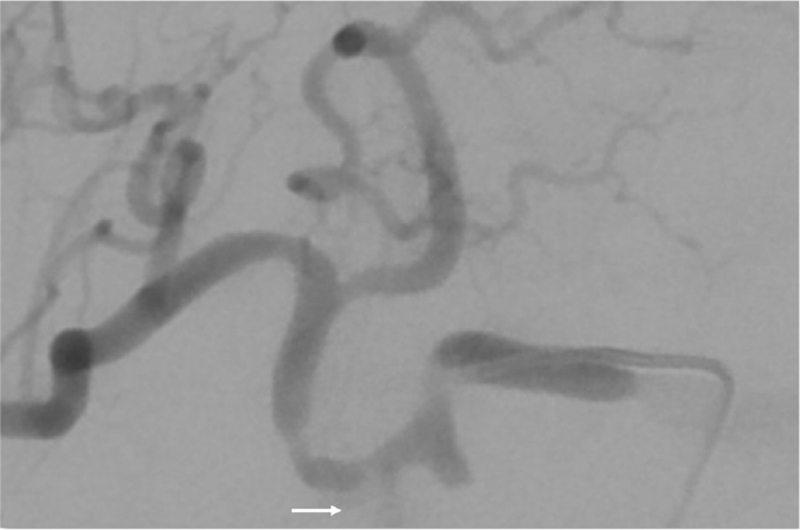
Arrow: Angiography showed extravasation of contrast from proper hepatic artery.

**Figure 4:**
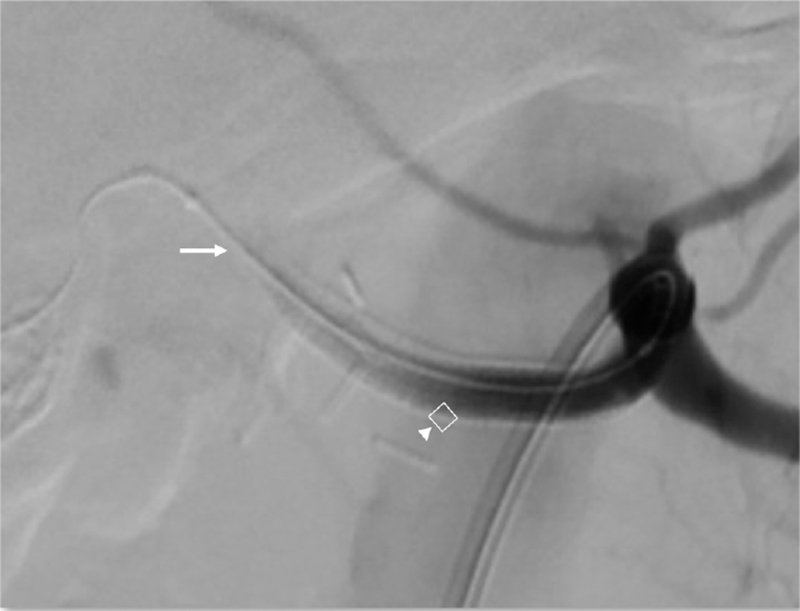
Arrow: No distal runoff after Viabahn 6 mm stent was placed, suspected intimal dissection. Arrowhead: Viabahn 6 mm stent.

**Figure 5:**
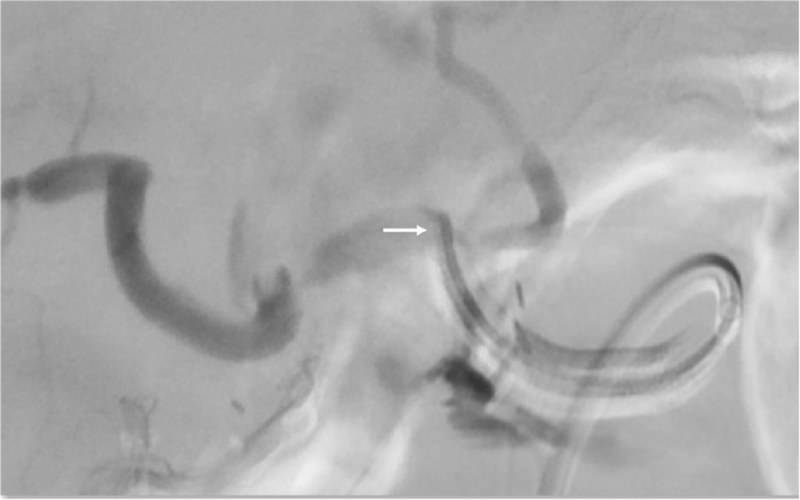
Angiography before deployment of 2^nd^ Viabahn stent to determine landing zone, 2 mm before bifurcation into right and left hepatic artery. Arrow: Bifurcation of right and left hepatic artery.

**Figure 6:**
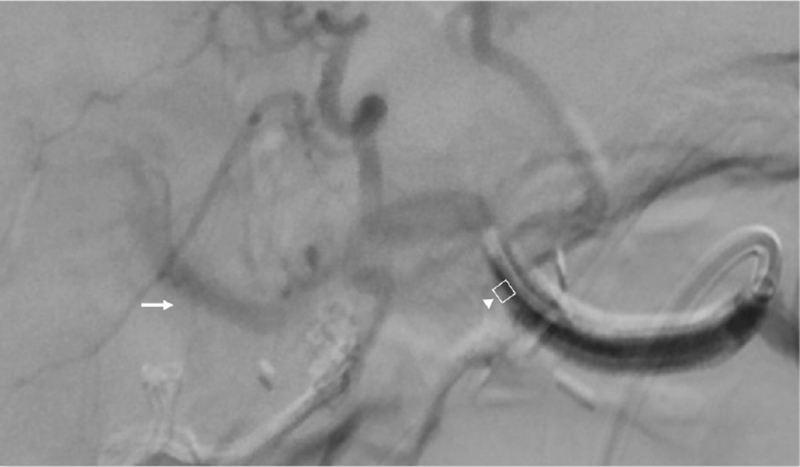
Arrow: Restore of hepatic artery flow after 2^nd^ Viabahn stent was deployed. Arrowhead: 2nd Viabahn stent.

This is a 74-year-old male with underlying chronic obstructive pulmonary disease, atrial fibrillation, presented with obstructive jaundice (total bilirubin 38 mg/dL). Computed tomography of abdomen showed a 2.16 × 1.92 cm enhancing nodule over ampulla vater region (Fig. 1), suspected malignancy causing distal common bile duct obstruction. Endoscopic retrograde cholangiography and pancreatography was carried out and 1 plastic stent 8.5 Fr × 12 cm was placed. Three weeks later, total bilirubin dropped to 2.8 mg/dL and pancreaticoduodenectomy with radical lymph node dissection was carried out. However, on postoperative day (POD) 2, pancreatic leak was found and treated with somatostatin line. Patient started enteral feeding on POD 5 but on POD 7 sentinel bleeding from drain was noted. Patient's hemoglobin level was 10.1 g/dL with no significant drop but slight hypotension (blood pressure 99/50 mm Hg). After fluid challenge, he was stabilized and computed tomography angiography was performed, revealing hemoperitoneum with no active extravasation (Fig. 2). Patient was transferred to hybrid operation room for angiography and proper hepatic artery active extravasation was noticed (Fig. 3). Wiring was then advanced distally and a 6 mm Viabahn stent was deployed to seal the bleeder but there was no distal runoff (Fig. 4). Due to compromised arterial true lumen, surgery was shifted to emergent laparotomy. Hepatic artery was divided at distal end of Viabahn stent and intimal dissection was noticed with previously placed stent within false lumen. Instead of artery ligation, modified VORTEC was carried out. Under direct visualization, intima and adventitia of hepatic artery was sutured together with 3 stitches of prolene 6-0. Guide wire was redirected into true lumen of distal hepatic artery and another 6 mm Viabahn stent was introduced with its proximal part overlying previous stent and distal part was pushed into true lumen of dissected distal hepatic artery about 2 cm. Before deploying the second stent, a soft intravenous catheter was placed over the opening of hepatic artery and contrast was injected to identify landing zone. It was then deployed before division of proper hepatic artery into right and left hepatic artery (Fig. 5). During the procedure, there was minimal bleeding from proximal side due to sheath placement. After ballooning of stent graft, 2 more sutures were done for reinforcement of stent graft to artery. Postdilatation angiography showed successful restoration of hepatic artery flow (Fig. 6) while Doppler showed normal waveform of both hepatic arteries. Total 8 units of 250 mL packed red blood cells were transfused and patient's condition stabilized when being transferred to intensive care unit. Immediately after operation, heparin was given and activated partial thromboplastin time kept between 1.5–2.5x with no consequent bleeding. Daily Doppler for a week showed intact arterial flow thus heparin was discontinued and shifted to clopidogrel to prevent in-stent stenosis. After 40 days of hospital stay, patient was successfully discharged without hepatic failure.

## 3 Discussions

Our case is a delayed, severe, extraluminal postpancreaticoduodenectomy bleeding of proper hepatic artery, most probably caused by Grade C pancreatic leak.^[1]^ Bleeding presented at POD 7 was a bit early, compared to other studies where most delayed hemorrhage usually presented at POD 13.^[2]^ However hypotensive episode suggested severe bleeding but was rapidly stabilized with fluid resuscitation. In choosing the optimal treatment, some studies advocate surgical treatment for unstable patients while radiological intervention for stable patients. However if PPDH is related to pancreatic fistula, adhesion might prevent early detection of bleeding site.^[3]^

Since patient's condition was hemodynamically stable, we opted for angiography in hybrid operation room. With angiography showing contrast extravasation from proper hepatic artery possible interventions included coil embolization or stenting, but in this case stent graft is our first choice as it helps preserve artery blood flow whilst stop extravasation, maintaining perfusion and reducing risk of end organ failure.^[4]^ However, after stenting was done, catastrophic complication occurred with intimal dissection of artery. Complications of endovascular management have been found in other case reports including rebleeding, stent stenosis, or thrombosis where most cases were successfully managed with repeated endovascular intervention.^[3]^ But this is not applicable to our case as true lumen of artery could not be located. Wiring or stent graft deployment may be the cause of dissection, thus we shifted to explore laparotomy to salvage the artery. Ligation of hepatic artery is the most effective way of bleeding control, however if artery flow were to be preserved, modified VORTEC is applicable.

**Figure 7:**
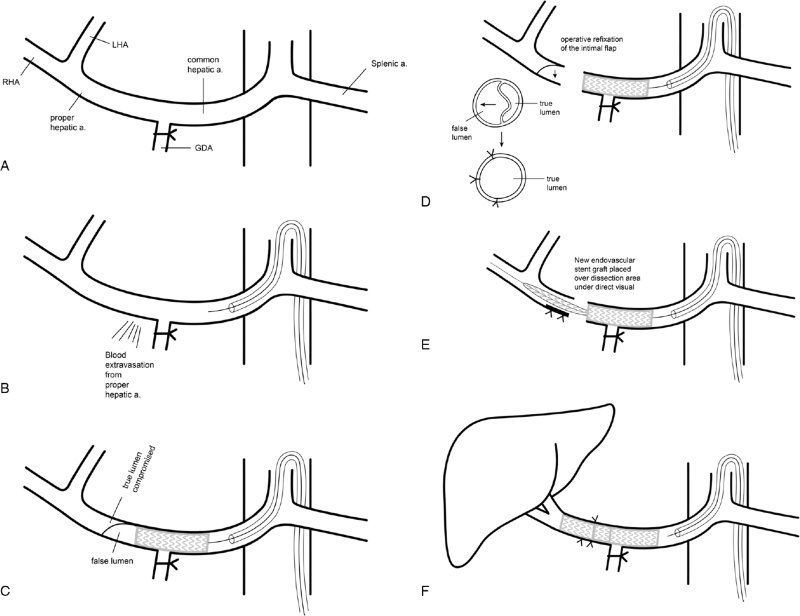
Illustration of Modified VORTEC procedure performed in our surgery. VORTEC = Viabahn Open Revascularization TEChnique.

VORTEC was first introduced for renal revascularization during aortic reconstruction in aneurysm surgery, but recently also applied in hybrid aortic arch surgery or in femoral bypass.^[5]^ We modified the original VORTEC where puncture is not required but with direct introduction of guidewire into target vessel and deployment of stent overlapping both sides of artery, reestablish blood flow (Fig. 7).^[6]^

This modified VORTEC has a few advantages. Its technical success rate is high because guidewire is introduced to the vessel under direct visualization. Furthermore, culprit vessels are more likely with fragile wall, possibly eroded or with aneurysm and stenting with Viabahn which is a flexible self-expanding stent with heparin bioactive surface, if successful, may provide better patency.^[7]^ Although blood flow is interrupted for a short term during intervention but the long-term result is preservation of end organ perfusion. Despite achieving technical success in this case, there are concerns regarding infection or in-stent stenosis. Due to direct introduction of guidewire between vessels, the stent and the vessels are both exposed to pancreatic or biliary juice, which may lead to severe infection. One of the issue with stent placement is possible stenosis or occlusion. Thus heparin line was initiated as soon as possible and later shifted to antiplatelet agent to prevent further stenosis.^[4]^

## 4 Conclusions

In summary, for management of patients with PPDH, endovascular treatment is the preferred choice in hemodynamic stable patients. Even though it is rather safe and minimally invasive, complications such as intimal dissection should be kept in mind. Currently, there is no literature describing VORTEC application on visceral surgeries and this is the first case illustrating its application in salvaging damaged vessels after initial endovascular treatment. We do not advocate its regular use for PPDH treatment as this strategy may not be applied to every patient and it requires advanced endovascular technique of an experienced cardiovascular surgeon in order to be carried out successfully. Nevertheless, this case demonstrates a possible bail-out management for life threatening bleeding and artery dissection.

## Acknowledgments

The authors wish to acknowledge Yuhan Keng in creating the wonderful illustration of this work.

## Author contributions

**Conceptualization:** Chee Chien Yong, Hsu-Ting Yen, Yi Ping Sng, Zhihao Li.

**Data curation:** Chee Chien Yong, Zhihao Li.

**Formal analysis:** Chee Chien Yong, Hsu-Ting Yen.

**Investigation:** Chee Chien Yong, Hsu-Ting Yen, Yi Ping Sng, Zhihao Li.

**Methodology:** Hsu-Ting Yen.

**Project administration:** Chee Chien Yong, Hsu-Ting Yen, Yi Ping Sng.

**Resources:** Chee Chien Yong, Yi Ping Sng.

**Software:** Yi Ping Sng.

**Supervision:** Chee Chien Yong, Hsu-Ting Yen.

**Validation:** Hsu-Ting Yen, Yi Ping Sng.

**Visualization:** Hsu-Ting Yen, Yi Ping Sng.

**Writing – original draft:** Yi Ping Sng, Zhihao Li.

**Writing – review & editing:** Chee Chien Yong, Yi Ping Sng, Zhihao Li.
